# Bacterial profile, treatment outcomes, and determinants among adult patients with musculoskeletal infections admitted to Jimma Medical Center: A prospective observational study

**DOI:** 10.1371/journal.pone.0322471

**Published:** 2025-05-02

**Authors:** Desalegn Gizaw, Gorfineh Teshome, Kisi Chemeda, Aster Wakjira, Mekonnen Damessa

**Affiliations:** Department of Clinical Pharmacy, Jimma University, Institute of Health, Jimma, Ethiopia; Southern Medical University Nanfang Hospital, CHINA

## Abstract

**Background:**

The epidemiological characteristics of musculoskeletal infections are constantly evolving. Despite the significant burden of musculoskeletal infections in sub-Saharan Africa, there is a scarcity of data regarding the potential causes and treatment outcomes of such infections in the region. Therefore, the primary objective of this study was to assess a management protocol, clinical outcomes, and associated factors among adult individuals diagnosed with musculoskeletal infections.

**Methods:**

Adult patients admitted to Jimma University Medical Center (JUMC), Ethiopia with a diagnosis of musculoskeletal infections was enrolled prospectively from July 2022-December 2022. Clinical characteristics, management protocol, and complications were recorded from admission to discharge. Bacteria were identified by a series of biochemical tests, and antimicrobial susceptibility testing was performed using the Kirby-Bauer Disk diffusion method. Data were analyzed by using SPSS v.23 and the p-value <0.05 was considered statistically significant.

**Result:**

Among 160 participants included in the study, 103(64.37%) were male, and the mean age 33.51 ± 13.56 years. About 94 (58.8%) of patients had microbial growth, of which 75.5% accounts for mono-microbial. *The common bacterial isolates were Staphylococcus aureus* in (22.4%), *Escherichia coli* (18.1%), *Pseudomonas aeruginosa* (14.7) and *Klebsiella pneumonia* (11.2). The isolated etiologies were resistant to Ceftriaxone in 67(81.70%) and Ceftazidime in 47(61.8%) of test results. Nearly all (98.8%) of patients were took antibiotics on admission and Ceftriaxone was highly utilized as monotherapy in (23.4%) of patients. Nearly half (46.9%) of the patients were develop treatment failure. Sex [AOR = 2.56, 95%CI (1.07–6.23)], microbial growth [AOR = 3.03, 95%CI (1.31–6.97)], ceftriaxone resistance [AOR = 4.46, 95%CI (1.06–18.67)], co-morbidities [AOR = 2.32, 95%CI (1.007–5.36)], and complications [AOR = 2.80, 95%CI (1.26–6.20)] were associated with treatment failure outcome.

**Conclusion:**

Close to half of adult patients with musculoskeletal infections experienced treatment failure. *Staphylococcus aureus* stood out as the prevailing bacterial strain identified. The timely switching of parenteral antibiotics to oral counterparts, alongside timely surgical interventions, significantly enhanced the recovery outcomes for these individuals.

## Introduction

Musculoskeletal infections (MSKIs), including osteomyelitis, septic arthritis, and soft tissue infections, are significant public health concerns, particularly in low-resource settings like sub-Saharan Africa[1–4]. These infections are associated with high morbidity, prolonged hospital stays, and substantial healthcare costs [5]. Despite the high burden of MSKIs in this region [6–9], there is a paucity of data on the bacterial profiles, antimicrobial resistance patterns, and treatment outcomes among adult patients. This gap in knowledge hampers the development of effective management protocols and contributes to poor clinical outcomes.

In Ethiopia, musculoskeletal infections are among the leading causes of hospital admissions [6,8], yet there is limited evidence on the etiological agents, their resistance patterns, and the factors influencing treatment outcomes. Previous studies in Ethiopia have either focused on pediatric populations [6] or were retrospective in nature [10], leaving a critical gap in understanding the bacterial profile and treatment outcomes among adult patients. Furthermore, the rising prevalence of antimicrobial resistance, particularly to commonly used antibiotics like ceftriaxone, underscores the need for updated and context-specific data to guide empirical treatment and improve patient outcomes.

This study aimed to address these gaps by prospectively investigating the bacterial profile, antimicrobial resistance patterns, treatment outcomes, and determinants of treatment failure among adult patients with musculoskeletal infections admitted to Jimma Medical Center, Ethiopia. By identifying the predominant pathogens, their resistance profiles, and the factors associated with treatment failure, this study provides critical insights that can inform local treatment guidelines and improve the management of MSKIs in similar settings.

The findings of this study are expected to contribute to the limited body of evidence on musculoskeletal infections in sub-Saharan Africa and provide a foundation for future research and policy interventions aimed at reducing the burden of these infections. Understanding the local epidemiology and resistance patterns is crucial for optimizing antibiotic use, reducing treatment failures, and improving patient outcomes in resource-limited settings

## Materials and methods

### Study design and population

The study was conducted at Jimma medical center (JMC), Jimma, Oromia, Ethiopia from July December 2022. JMC is located in Jimma town, situated around 352 kilometers southwest of Addis Ababa. Jimma Medical Center has been delivered services for approximately 20,000 inpatients, 160,000 outpatients, 11,000 emergency cases and 4,500 deliveries in a year. Additionally, JMC have a total of 20 wards with 671 beds; also, the study was carried out at Orthopedics, surgery, and medical wards. There are 40 beds in orthopedics, 78, and 86 beds in surgery and medical ward, respectively. Orthopedics ward and Surgery ward render service under Surgery department. Facility based prospective observational study was conducted [11].

All patients with signs and symptoms of musculoskeletal infections and fulfilling the inclusion criteria were enrolled by treating physician and confirmed through clinical examination. Patients with confirmed diagnosis of MSKIs, Patients who were willing to participate in the study were the inclusion criteria; while patients having laboratory values that are non-conclusive (unidentified microorganism report), patients who were died or discharged before isolation of the microorganism and initiation of treatment were excluded during study.

### Data collection

The data collection instrument was crafted through a comprehensive review of previous studies conducted across various contexts. It encompasses sociodemographic details, clinical features (encompassing laboratory tests and essential diagnostic assessments), treatment approaches (including medications and surgical procedures), and in-hospital results (comprising complications, duration of hospitalization, and discharge clinical condition).

Sociodemographic characteristics such as age, sex, residence and educational status, were recorded by interviewing patients. Clinical features, laboratory results, treatment modalities and treatment outcomes were collected from patients’ medical chart.

### Specimen collection, handling, and transport

#### Identification and antimicrobial susceptibility testing (AST).

Specimen obtained from the patients (Blood, Pus, Wound, Drainages, and Abscesses) by senior nurse until the swab became wet purulent discharge and for blood specimen 8-10 ml/bottle of blood were drawn bilaterally from both hands on the properly labelled two bottles. Within 2 hours of specimen collection, it was delivered to the microbiology laboratory then the samples were inoculated onto blood agar, Chocolate agar, and MacConkey agar. Then the plate was incubated for 18- 24 hours at 35-37^o^C MacConkey Agar Plate (MAP) aerobically and Blood Agar Plate (BAP) under CO2. If there was no growth after 18-24 hours of incubation, record the preliminary result & re-incubate BAP for additional 18-24 hours. When there was growth after 18-24 hours of incubation, a definitive biochemical identification and antimicrobial susceptibility test was performed. Standard operating procedures were followed in specimen collection and handling [12].

#### Identification of bacteria.

Each specimen was inoculated onto MacConkey agar (MAC), mannitol salt agar (MAS), and blood agar plate (BAP), and chocolate agar plate (CAP) culture media. The media were incubated at 37 °C for 24 and 48 hours and aerobic conditions were maintained. For fastidious organisms, chocolate agar (heated 5% sheep’s blood agar) was incubated at 37 °C for 24–48 hours in a 5–10% CO2 atmosphere [12,13]. All plates were examined for bacterial growth after 24 hours and plates with no bacterial growth were reincubated for another 24 hours. After obtaining pure colonies, specific bacterial pathogens were identified by Gram stain, colony morphology, and a series of biochemical tests. Catalase, coagulase, optochin disk sensitivity, novobiocin, and bacitracin tests were conducted to identify gram-positive bacteria. Biochemical tests such as lysine decarboxylase, citrate utilization, lactose fermentation, indole, urease, oxidase and satellitism tests were used to identify gram-negative bacteria.

#### Antimicrobial susceptibility test.

Antimicrobial susceptibility test was carried out by using the Kirby Bauer (KB) disk diffusion method, then a standardized inoculum of the bacteria was swabbed onto the surface of a Mueller Hinton agar (MHA) plate. Three to five similar colonies were picked up with a wooden applicator stick and dipped into normal saline to make direct colony suspension of the isolates and inoculum was adjusted at 0.5 McFarland standard by using a densitometer.

The antibiotic susceptibility testing was done for Ampicillin (10µg), Amikacin (30µg), Ampicillin-sulbactam (10/10µg), Gentamicin (10µg), Ceftriaxone (30µg), Ciprofloxacin (5µg), Trimethoprim-Sulphamethoxazole(1.25/23.75µg), Ceftazidime (30 µg), Clindamycin, Cefepime (30 µg), Amoxicillin-Clavulanic acid (10µg), Meropenem (10 µg), Vancomycin, Cloxacillin, Cephalexin and Chloramphenicol. The plates were incubated at 37 °c for 24 hours under aerobic conditions and the diameter of inhibition zones was measured using a ruler and compared with the standard set by CLSI. Quality control of the susceptibility testing was assured by testing strains of *E. coli* ATCC 25922 for *Enterobacteriaceae, P. aeruginosa* ATCC 27853 Non- (*Enterobacteriaceae*), and Gram Positive *S.aureus* ATCC 25923 [14]. Multidrug resistance was considered if bacterial isolates were resistant to three or more antibiotics from different classes.

### Data quality control and management

The data collectors were tutored on the goal of the study, the parts of the questionnaires, their responsibility, and obligations. The questionnaire was translated from English to Amharic and afaan Oromo and back to English. Sociodemographic characteristics and clinical data were collected by residents and all specimens were collected following CLSI guidelines [12]. The sterility of culture media was ensured by incubating 5% of each batch of the prepared media at 37 °C for 24 hours. To preserve the quality of the results, physical changes including cracks, excess moisture, changes in color, and dehydration, were checked routinely. The incubator and refrigerator temperatures were monitored on a daily basis. The quality and performance of culture media and biochemical tests were checked using standard strains, such as *S. aureus* (ATCC 25923), *E. coli* (ATCC 25922), and *P. aeruginosa* (ATCC 27853).

### Data processing and analysis

The collected data were checked for completness and entered into Epi data version 4.6. Then, the data were imported and analyzed using IBM SPSS software (Version 27:0; IBM SPSS Inc., New York, USA). A chi-square (χ2) test was done to assess the associations between categorical variables. Descriptive statistical analysis was employed to provide an overview of the patients’ baseline characteristics. A normality test was conducted using Shapiro-Wilk’s W test for continuous data, and outcome variables were described with mean ± standard deviation. Categorical variables were presented by frequency and percentage. Bivariate and multivariate logistics regression models were used to determine the presence of an association between independent and dependent variables. Variables with a P value <0.25 were candidates for stepwise multivariate analysis. In multivariate analysis, the adjusted odds ratio was used to measure the strength of association and variables with p-value <0.05 were considered statistically significant.

### Outcome measure and validating methods

The primary outcome of the present study was in-hospital treatment outcomes which were assessed by following the patient’s clinical signs and symptoms and occurrence of complications until discharge. The treatment outcomes in this study were categorized as either fully recovered or treatment failure. Patients were classified as treatment failure if they exhibited local or systemic signs of infection, reinfection, disease progression, delayed wound healing, or complications requiring additional procedures after completing the initial treatment course. Conversely, patients were classified as fully recovered if none of these symptoms or complications were present upon completion of their treatment course. The treating physician confirmed in-hospital complications based on clinical findings and objective evidence like culture, ultrasound, and X-ray imaging. Additionally, the treating physicians reviewed discharge summary notes and monitored patients daily from admission to discharge.

### Ethical consideration

The study protocol was approved by the Institutional Review Board (IRB) of Jimma University, Institute of Health with a Ref. No IHRP1/1212/22. Approval was sought from the appropriate hospital authorities prior to interviewing the patients and extracting data from the patients’ charts. Furthermore, both written and verbal informed consent was obtained from all participants before initiating the data collection process. All the study protocols were performed in accordance with the ethical principles of the Declaration of Helsinki [15].

## Results

### Participant’s enrollment information

**Fig 1:**
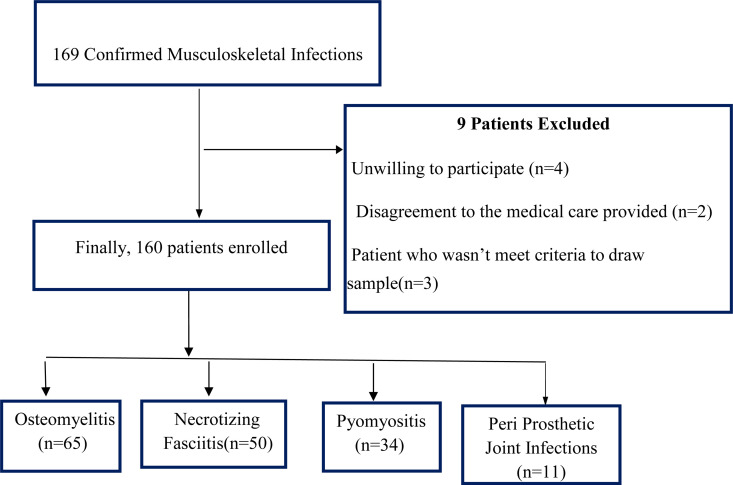
Flow diagram of Participant enrollments.

Throughout the study duration, a collective of 169 patients with MSKIs were recognized from the Orthopedics, Surgery, and Internal Medicine departments at JMC. Of these, nine patients were excluded, leaving 160 individuals who were enrolled, monitored, and incorporated into the analysis (Fig 1).

### Socio-demographic characteristics

Among 160 patients with MSKIs included in the study, the majority 103(64.4%) of the participants were male. The mean ± SD age of patients was 33.51±13.56 years. About two-thirds (63.7%), of the patients were rural residents. Regarding educational status, 59(36.9%) of patients could not read and write, while farmers were common occupational status in 42(26.3%), followed by soldiers 28(17.5) (Table 1).

**Table 1:** Socio-demographic characteristics of patients with musculoskeletal infections admitted to JMC, July- December 2022.

Variables Category		Types of Musculoskeletal infections				
		OM n (%)	Necrotizing fasciitis n (%)	Pyomyositis n (%)	PJI n (%)	Total n (%)
Sex	Male	44(67.7)	31(62)	19(55.9)	9(81.8)	103(64.4)
	Female	21(32.3)	19(38)	15(44.1)	2(18.2)	57(35.6)
Body Mass Index (BMI)(Kg/m^2^)	Underweight (Below 18.5)	9(13.8)	6((12)	3(8.82)	1(9.1)	19(11.9)
	Healthy Weight (18.5–24.9)	48(73.9)	41(82)	24(70.59)	10(90.9)	123(76.9)
	Overweight (25.0–29.9)	8(12.3)	3(6)	7(20.59)	–	18(11.3)
Residence	Rural	26(40)	14(28)	12(35.3)	6(54.5)	102(63.7)
	Urban	39(60)	36(72)	22(64.7)	5(45.5)	58(36.3)
Educational background	Cannot Read and Write	18(27.7)	26(52)	11(32.4)	4(36.4)	59(36.9)
	Primary School	22(33.9)	12(24)	15(44.1)	–	49(30.6)
	Secondary School	16(24.6)	8(16)	3(8.8)	2(18.2)	29(18.1)
	College and above	9(13.8)	4(8)	5(14.7)	5(45.4)	23(14.4)
Occupation	Farmer	10(15.4)	16(32)	7(20.6)	2(18.2)	42(26.3)
	Merchant	9(13.9)	4(8)	2(5.9)	–	15(9.4)
	Government employee	4(6.2)	3(6)	2(5.9)	1(9.1)	14(8.8)
	Housewife	6(9.2)	11(22)	5(14.7)	–	22(13.8)
	Laborer	4(6.2)	3(6)	6(17.6)	–	13(8.1)
	Driver	7(10.7)	3(6)	–	1(9.1)	11(6.9)
	Non Gov.Employee	7(10.7)	4(8)	4(11.8)	–	15(9.4)
	Soldier	18(27.7)	6(12)	8(23.5)	7(63.3)	28(17.5)
Age (Mean ± SD)					33.51 ± 13.56	

### Clinical features and related factors

On admission, all patients with MSKIs manifested localized signs and symptoms; swelling at the site of infection in 150(93.8%) and tenderness in 128(80%) were identified as a clinical presentation of the infections (Table 2)

**Table 2:** Baseline clinical finding of the study participants who were diagnosed with MSKIs admitted to JMC, July- December 2022.

Variables	Category	Frequency(n)	Percent (%)
Temperature on admission	Hyperthermia	96	60.0
	Normothermia	58	36.3
	Hypothermia	6	3.8
Localized signs and symptoms	Yes	160	100.0
Swelling	Yes	150	93.8
	No	10	6.3
Tenderness	Yes	128	80.0
	No	32	20.0
Warmth at the site	Yes	97	60.6
	No	63	39.4
Erythema	Yes	114	71.3
	No	46	28.8
Pain on admission	Yes	153	95.6
	No	7	4.4
Difficulty in range of limb movement	Yes	153	95.6
	No	7	4.4
Difficulty in weight-bearing	Yes	141	88.1
	No	19	11.9
Abscess formation	Yes	80	50.0
	No	80	50.0

### Laboratory investigation and imaging-related factors

Complete blood count (CBC) was done for all patients on admission; of which leukocytosis and neutrophils were elevated in 106(66.3%) and 114(71.3%) of patients respectively. Similarly, C reactive protein was done for 69(43.1%) of patients, and the value was high in 63(91.3%), while Erythrocyte Sedimentation Rate in 141 (88.1%) and the value was high in 129(91.5%) of patients. Imaging results, x-ray in 150 (93.8%) and ultrasound in 80(50%) were also obtained as baseline diagnostic tools (Table 3).

**Table 3:** Baseline laboratory and imaging findings among patients with MSKIs admitted to JMC, July- December 2022.

Variables	Category	Frequency(n)	Percent (%)
CBC test on admission	Yes	160	100.0
	No	–	–
White Blood Cell (cell/ mm3)	High	106	66.3
	Normal	54	33.8
Neutrophil (cell/ mm3)	High	114	71.3
	Normal	46	28.8
Red Blood Cell (cell/ mm3)	High	6	3.8
	Normal	102	63.8
	Low	52	32.5
Hemoglobin (g/dl)	Normal	95	59.4
	Low	65	40.6
Hematocrit (%)	Normal	96	60.0
	Low	64	40.0
Mean Corpuscular Volume(pg/dl)	High	5	3.1
	Normal	116	72.5
	Low	39	24.4
Platelet (cell/ mm3)	Thrombocytosis	39	24.4
	Normal	99	61.9
	Thrombocytopenia	22	13.8
C reactive protein (mg/dl)	Yes	69	43.1
	No	91	56.9
Value of CRP	High	63	91.3
	Normal	6	8.7
Erythrocyte Sedimentation Rate (mm/hr)	Yes	141	88.1
	No	19	11.9
Value of ESR	High	129	91.5
	Normal	12	8.5
Synovial Fluid Analysis	Yes	11	6.9
	No	149	93.1
Ultrasound	Yes	80	50.0
	No	80	50.0
X-ray	Yes	150	93.8
	No	10	6.3

### Bacterial profile and related factors

Culture was done for all patients with MSKIs and; microbial growth was identified in 94(58.8%) of the patients. Monomicrobial was a common microorganism profile identified in 71(75.7%) patients who had bacterial growth. *Staphylococcus aureus* 26(22.4%) was a main etiology isolated among patients with MSKIs followed by gram-negative etiologies *Escherichia coli* in 21(18.1%) and *Pseudomonas aeruginosa* in 17(14.7%) (Table 4).

**Table 4:** Distribution of microorganisms identified among participants who were diagnosed with MSKIs admitted to JMC, July- December 2022.

Variables	Category	Frequency(n)	Percent (%)
Culture on Admission	Yes	160	100.0
	No	–	–
Microbial Growth	Yes	94	58.8
	No	66	41.3
Number of bacterial isolates	Monomicrobial	71	75.5
	Polymicrobial	23	24.5
Bacterial profiles	*Staphylococcus aureus*	26	22.4
	*Klebsiella pneumonia*	13	11.2
	*Pseudomonas aeruginosa*	17	14.7
	*Escherichia coli*	21	18.1
	*Coagulase Negative Staphylococci (CoNS)*	6	5.2
	*Proteus Mirabilis*	11	9.5
	*Citrobacter Species*	10	8.6
	*Enterococcus. Species*	3	2.6
	*Enterobacter species*	3	2.6
	Others^*^	6	5.2

* Others: *Streptococcus Pneumonae*, *Acinetobacter Species*, *Proteus Vulgaris*, and *Salmonella Species*

### Antimicrobial susceptibility test-related factors

A susceptibility test was done for isolated microorganisms and reported that majority of isolated etiologies were resistant to Ceftriaxone 67(81.7%), Ceftazidime 47(61.8%) and Gentamicin 41 (46.5%) of test results. However, Clindamycin 6(20%) and Vancomycin 1(6.7%) were among the antibiotics with a low resistant profile in the setting (Table 5).

**Table 5:** Antimicrobial susceptibility patterns of isolates identified from patients presented with musculoskeletal infections admitted to JMC, July- December 2022.

Bacterial Isolate	Total	Paterns	Antimicrobial agents tested													
			AMP f (%)	AMC f (%)	CEF f (%)	CAZ f (%)	CAF f (%)	CIP f(%)	CLI f (%)	ERY f(%)	GEN f(%)	MER f (%)	PEN f (%)	PIP f (%)	TMX f (%)	VAN f (%)
*S.aureus*	26	S	1(20)	–	1(10)	1(14.3)	8(88.9)	10(50)	16(72.7)	8(34.8)	10(71.43)	5(71.4)	5(20.8)	3(42.86)	5(29.4)	10(100)
		I	–	1(16.7)	–	–	–	4(20)	1(4.6)	7(30.4)	1(7.14)	–	–	2(28.57)	2(11.8)	–
		R	4(80)	5(83.3)	9(90)	6(85.7)	1(11.1)	6(30)	5(22.7)	8(34.8)	3(21.43)	2(28.6)	19(79.2)	2(28.57)	10(58.8)	–
*K. pneumonia*	13	S	1(14.3)	1(10)	2(15.38)	2(18.2)	2(22.2)	–	–	–	4(36.4)	7(70)	–	9(81.8)	1(12.5)	–
		I	–	4(40)	–	–	–	6(66.7)	–	–	–	–	–	–	–	–
		R	6(85.7)	5(50)	11(84.62)	9(81.8)	7(77.8)	3(33.3)	–	–	7(63.6)	3(30)	2(100)	2(18.2)	7(87.5)	–
*P.auroginosa*	17	S	–	2(28.57)	3(25)	9(56.25)	3(33.3)	6(42.86)	2(66.7)	–	4(30.77)	12(75)	–	5(35.72)	9(52.9)	3(100)
		I	–	3(42.86)	–	5(31.25)	4(44.5)	1(7.14)	–	–	3(23.08)	–	–	8(57.14)	1(5.9)	–
		R	2(100)	2(28.57)	9(75)	2(12.5)	2(22.2)	7(50)	1(33.3)-	–	6(46.15)	4(25)		1(7.14)	7(41.2)	–
*E. coli*	21	S	–	7(50)	7(38.9)	3(18.75)	11(78.57)	3(18.75)	–	–	10(47.62)	16(88.9)	–	11(61.1)	2(13.3)	–
		I	–	5(35.7)	–	3(18.75)	–	3(18.75)	–	–	–	–	–	3(16.7)	**–**	–
		R	15(100)	2(14.3)	11(61.1)	10(62.5)	3(21.43)	10(62.5)	–	–	11(52.38)	2(11.1)	–	4(22.2)	13(86.7)	–
*CoNS*	6	S	1(16.7)	2(50)	–	1(16.7)	–	2(40)	2(100)	2(66.7)	3(50)	4(66.7)	2(33.3)	2(33.3)	3(60)	2(100)
		I	1(16.7)	–	–	2(33.3)	–	2(40)	–	1(33.3)	–	–	–	–	–	–
		R	4(66.6)	2(50)	5(100)	3(50)	–	1(20)	–	–	3(50)	2(33.3)	4(66.7)	4(66.7)	2(40)	–
*P.mirabilis*	11	S	–	1(14.2)	1(10)	2(25)	5(45.5)	1(12.5)	–	–	3(33.3)	9(81.8)	–	5(55.6)	2(22.2)	–
		I	–	3(42.9)	–	–	–	1(12.5)	–	–	2(22.2)	–	–	2(22.2)	–	–
		R	9(100)	3(42.9)	9(90)	6(75)	6(54.5)	6(75)	–	–	4(44.5)	2(18.2)	–	2(22.2)	7(77.8)	–
*Citrobacter Sp.*	10	S	–	1(12.5)	1(12.5)	1(14.3)	4(50)	1(12.5)	–	–	3(33.3)	8(80)	–	4(50)	1(14.3)	–
		I	–	4(50)	–	–	–	1(12.5)	–	–	1(11.1)	–	–	3(37.5)	–	–
		R	7(100)	3(37.5)	7(87.5)	6(85.7)	4(50)	6(75)	–	–	5(55.6)	2(20)	–	1(12.5)	6(85.7)	–
*Enterococcus Sp.*	3	S	–	1(50)	–	–	2(66.7)	1(33.3)	3(100)	2(100)	2(66.7)-	–	1(33.3)	–	–	2(66.7)
		I	–	–	–	–	–	–	–	–	–	–	–	–	–	–
		R	–	1(50)	3(100)	3(100)	1(33.3)	2(66.7)	–	–	1(33.3)	3(100)	2(66.7)	3(100)	3(100)	1(33.3)
*Enterobacter sp.*	3	S	–	–	–	1(33.3)	2(66.7)	–	–	–	1(33.3)	3(100)	–	1(50)	–	–
		I	–	2(66.7)	–	–	1(33.3)	–	–	–	–	–	–	1(50)	–	–
		R	–	1(33.3)	3(100)	2(66.7)	–	2(100)	–	–	2(66.7)	–	–	–	3(100)	–

AMP: Ampicillin, AMC:Augmentin, CEF: Ceftriaxone, CAZ: Ceftazidime, CAF: Ciprofloxacin, CLI:Clindamycin, ERY: Erythromycin, GEN: Gentamycin, MER: Meropenem, PEN: Penicillin, PIP: Piperacillin/tazobactam, TMX: Trimethoprim/sulfamethoxazole, VAN: Vancomycin

### Disease-related factors

#### Types of musculoskeletal infections.

**Fig 2:**
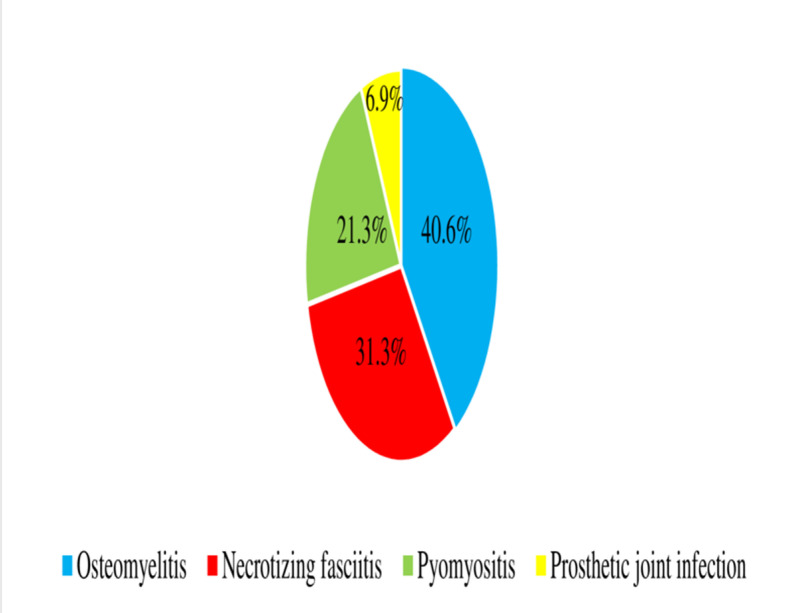
Distributions of musculoskeletal infections type among patients with MSKIs admitted to JMC.

Osteomyelitis in 65 (40.6%), necrotizing fasciitis in 50 (31.3%), and Pyomyositis in 34(21.3%) were the predominant types of MSKIs identified among the studied participants (Fig 2).

#### Anatomical locations affected by MSKIs.

The most commonly affected anatomical site was the tibia in 57(34.1%) of patients, followed by Femur in 49(29.3%) (Table 6).

**Table 6:** Anatomical location of musculoskeletal infection among participants admitted to JMC, July- December 2022.

Variables	Category	Frequency(n)	Percent (%)
Anatomical area affected by MSKIs	Tibia	57	34.1
	Femur	49	29.3
	Elbow	14	8.4
	Humerus	12	7.2
	Foot	11	6.6
	Knee	10	6.0
	Ankle	9	5.4
	Hip	5	3.0

### Risk factors of MSKIs

Trauma 93(58.1%), and comorbidities 82(51.2%) were the common risk of MSKIs in the study area. The mean ± SD of symptom duration before the patient was admitted to the setting was 32 ± 53.70 days. For patients who had comorbidities, diabetes mellitus 29(27.9%), cardiac disease 23(22.1%), and tuberculosis 14(13.5%) were the common comorbidities, almost one-fourth used immunosuppressive drugs (Table 7).

**Table 7:** The risk factors of MSKIs among patients admitted to JMC, July- December 2022.

Variables	Category	Frequency(n)	Percent (%)
Cause for MSKIs(n = 160)	Trauma	93	58.1
	Hematogenous spread	67	41.9
Symptom duration (Mean ± SD)	53.70 ± 32 days		
Presence of Comorbidity (n = 160)	Yes	82	51.2
	No	78	48.8
Comorbidities Type (n = 104)	Diabetes mellitus	29	27.9
	Cardiac Disease	23	22.1
	HIV	8	7.7
	Tuberculosis	14	13.5
	Malnutrition	11	10.6
	Cancer	5	4.8
	Liver Disease	8	7.7
	Chronic kidney disease	6	5.8
Immune suppressive drug(n = 82)	Yes	23	28.0
	No	59	72.0
Types of immune suppressive drug	Chemotherapy Drugs	7	30.4
	Steroids	16	69.6
Antibiotic Exposure before admission	Yes	63	39.4
	No	97	60.6
Antibiotics Used Before admission (n = 83)	Ceftriaxone	53	63.9
	Metronidazole	19	22.9
	Cloxacillin	5	6.0
	Ciprofloxacin	6	7.2

### Treatment-related factors

#### Medical management of musculoskeletal infections.

Nearly all 158(98.8%) patients took antibiotics on their admission. Over one-fourth (25.9%) of patients who took antibiotics were treated with a monotherapy regimen. Ceftriaxone was a predominant antibiotic utilized as monotherapy in 37(23.4%) of patients; whereas Ceftriaxone plus metronidazole was a common combination of antibiotics in 50(42.4%) followed by Ceftriaxone plus Gentamicin in 28(23.7%) of patients. Almost all 157(98.1%) of patients took parenteral antibiotics and the majority 49(31.0%) of the patients were on parental antibiotics for five to six weeks.

In sixty-nine (43.7%) patients, parenteral antibiotic was shifted to oral antibiotic. The majority of patients, 23(33.3%) switched PO antibiotics after 4 weeks of treatment. Ciprofloxacin 23 (33.8%) and Cloxacillin 12 (16.2%) were common PO antibiotics utilized among patients with MSKIs.

Additionally, antipain medications were considered for 139 (86.9%) of the patients. Tramadol118 (50.2%) and Diclofenac 72(30.6%) were predominantly considered to treat pain for these patients (Table 8).

**Table 8:** Medication use characteristics among adult patients with MSKIs admitted to JMC, July- December 2022.

Variables	Category	Frequency(n)	Percent (%)
Antibiotics on admission	Yes	158	98.8
	No	2	1.3
Number of antibiotics	Monotherapy	41	25.9
	Combination therapy	117	74.1
Initial Route of Drug Administration	Intravenous	157	98.1
	PO	1	0.6
Antibiotics Used	Ceftriaxone Monotherapy	37	23.4
	Ceftriaxone + Metronidazole	50	42.4
	Ceftriaxone + Gentamicin	28	23.7
	Ceftriaxone +Metronidazole + Gentamicin	18	15.3
	Ceftazidime + Vancomycin	12	10.2
	Ceftriaxone + Gentamicin + Clindamycin	10	8.5
Duration of IV* antibiotics	Less than 1 Week	2	1.3
	For 1–2 Weeks	35	22.2
	For 3–4 Weeks	58	36.7
	For 5–6 Weeks	49	31.0
	For 7–8 Weeks	14	8.9
Switched to PO** antibiotics	Yes	69	43.7
	No	89	56.3
Time taken to switch IV to PO**	< 1 Week	1	1.4
	After 1 week of treatment	10	14.5
	After 2 weeks of treatment	13	18.8
	After 3 weeks of treatment	22	31.9
	After 4 weeks of treatment	23	33.3
PO** antibiotics After Switching	Ciprofloxacin	25	33.8
	Cephalexin	9	12.2
	Chloramphenicol	8	10.8
	Amoxicillin/Clavulanate	9	12.2
	Trimethoprim-sulfamethoxazole	8	10.8
	Cloxacillin	12	16.2
	Others***	3	4.2
Anti-pains Used	Yes	139	86.9
	No	21	13.1
Types of anti-pain medications	Tramadol	118	50.2
	Morphine	31	13.2
	Pethidine	1	0.4
	Diclofenac	72	30.6
	Paracetamol	13	5.5

(PO**: per oral or by mouth, IV*: Intravenous, Others***: Metronidazole, Clindamycin, and Erythromycin)

### Surgical management

One hundred forty-two (88.8%) of patients were undergone surgical interventions as treatment of these infections. surgical incision and drainage in 97 (40.2%) and debridement in (26.1%) were common surgical procedures performed for those patients (Table 9).

**Table 9:** Types of surgical procedures performed and length of hospital stay among adult patients with MSKIs admitted to JMC, July- December 2022.

Variables	Category	Frequency(n)	Percent (%)
Surgical Intervention	Yes	142	88.8
	No	18	11.2
Types of Surgical Procedures	Incision and drainage	97	40.2
	Debridement of Infected Tissues	63	26.1
	Irrigation	22	9.1
	Sequestrectomy	19	7.9
	Removal of infected prostheses	11	4.6
	Skin Grafting	8	3.3
	Amputation	8	3.3
	Arthrotomy	7	2.9
	Prosthetic Device Retention	6	2.5
LOS(mean ± SD in days)	38.74 ± 13.17		

#### Treatment outcomes.

**Fig 3:**
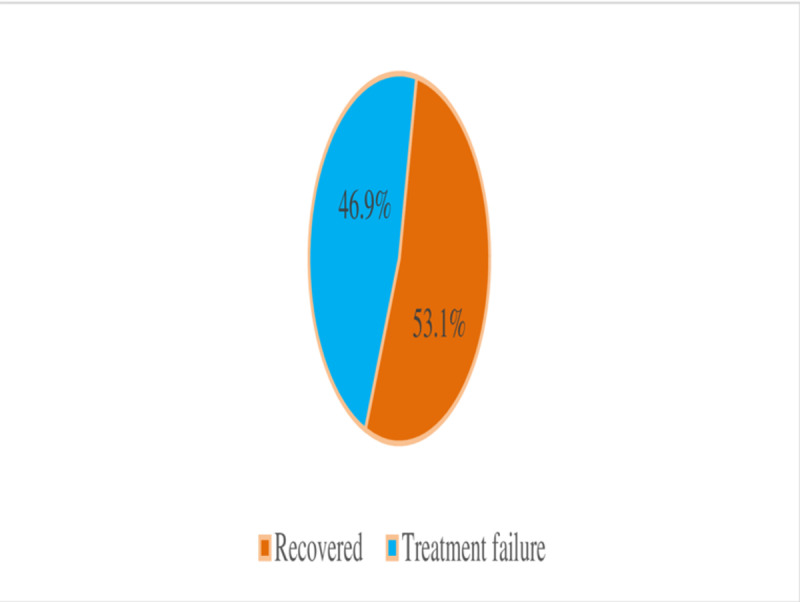
Treatment outcomes of musculoskeletal infections among participants admitted to JMC.

Treatment failure was observed in 75 (46.9%) of the patients treated for MSKIs in the setting (Fig 3).

#### In-hospital complications of musculoskeletal infections.

**Fig 4:**
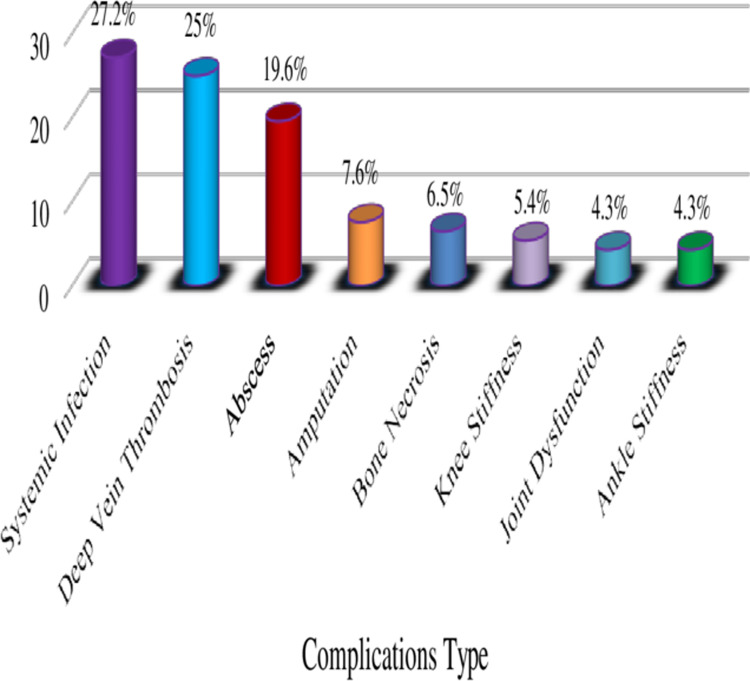
Distributions of in-hospital complications among adult patients with MSKIs.

Of the enrolled participants, 75 (46.9%) of the patients had developed at least one in-hospital complication during the study. the most common types of complications were: systemic infections (27.2%), deep vein thrombosis (25%), and abscesses (19.6%) were the most common in-hospital complications identified among these patients (Fig 4).

#### Factors associated with MSKIs treatment outcomes.

Out of 160 patients treated for MSKIs, 75 (46.9) patients had experienced treatment failure. On multivariate logistic regressions male sex [AOR = 2.56, 95%CI (1.07–6.23), P = 0.034], having of microbial growth [AOR = 3.03, 95%CI (1.31–6.97), P = 0.009], ceftriaxone resistance [AOR = 4.46, 95%CI (1.06–18.67), P = 0.040], having co-morbidities [AOR = 2.32, 95%CI(1.007–5.36), P = 0.048], and Presence of complication [AOR = 2.80, 95%CI (1.26–6.20), P = 0.011] were predictors of treatment failure; whereas switching of antibiotics [AOR = 0.30, 95%CI (0.13–0.67), P = 0.004] and having surgical interventions [AOR = 0.09, 95%CI (0.01–0.44), P = 0.003] were protective against treatment failure (Table 10).

**Table 10:** Bivariate and Multivariate Analysis of factors associated with treatment outcomes among patients with MSKIs admitted to JMC July- December 2022.

Variables	Categories	Treatment outcomes		COR (95% CI)	p-value	AOR (95%) CI	P-value
		Treatment Failure n (%)	Recovered n (%)				
Sex	Male	55(53.4)	48(46.6)	2.12(1.08-4.13)	0.027	2.56(1.07-6.23)	**0.034** ^*^
	Female	20(35.1)	37(64.9)	1	–	–	–
Microbial Growth	Yes	56(59.6)	38(40.4)	3.64(1.86-7.15)	0.001	3.03(1.31-6.97)	**0.009** ^*^
	No	19(28.8)	47(71.2)	1	–	–	–
Home Residency	Rural	56(54.9)	46(45.1)	2.49(0.42-7.9)	0.008	2.02(0.82-4.93)	0.122
	Urban	19(32.8)	39(67.3)	1	–	–	–
Ceftriaxone resistance	Sensitive	5(33.3)	10(66.7)	1	–	–	–
	Resistance	45(67.2)	22(32.8)	4.09(1.24-13.42)	0.020	4.46(1.06-18.67)	**0.040** ^*^
Co-morbidity	Yes	47(57.3)	35(42.7)	2.39(1.26-4.53)	0.007	2.32(1.007-5.36)	**0.048** ^*^
	No	28(35.9)	50(64.1)	1	–	–	–
Diabetes mellitus	Yes	21(72.4)	8(27.6)	3.74(1.54-9.07)	0.003	1.13(0.11-11.28)	0.91
	No	54(41.2)	77(58.8)	1	–	–	–
IV to PO Switching	Yes	22(31)	49(69)	0.30(0.15-0.59)	0.001	0.30(0.13-0.67)	**0.004** ^*^
	No	53(59.5)	36(40.5)	1	–	–	–
Surgical Intervention	Yes	60(42.3)	82(57.7)	0.14(0.04-0.52)	0.003	0.09(0.01-0.44)	**0.003** ^*^
	No	15(83.3)	3(16.7)	1	–	–	–
Complication	Yes	51(60)	34(40)	3.18(1.66-6.11)	0.001	2.80(1.26-6.20)	**0.011** ^*^
	No	24(32)	51(68)	1	–	–	–

## Discussions

This study assessed the bacterial profile, drug susceptibilities, management, treatment outcomes, and its determinants among patients with musculoskeletal infections.

The current study reported that *S. aureus* was the most commonly isolated organism in the setting, followed by gram-negative etiologies *Escherichia coli* (18.1%) and *Pseudomonas aeruginosa* (14.7%). This finding is similar to other existing literatures conducted in Sweden [16], USA(17), South Africa [5], India [17], Nigeria [18] and Republic of China [19]. However, different finding was reported from Republic of China [20], and two studies from France [21,22]. The difference might be due to variations in geographical location, anatomical site, and comorbidity (in the study of China, patients with sepsis fungal etiology are common and in study of France, patients diagnosed with peripheral neuropathy and peripheral arterial disease, also *Streptococcus species* are common).

The majority of isolated etiologies were resistant to Ceftriaxone (81.70%), Ceftazidime (61.8%) and Penicillins (77.1%). However, Clindamycin (20.0%) and Vancomycin (6.7%) were among the antibiotics with low resistant profiles in the setting. In contrast, study conducted in Germany [23], the antibiotic susceptibilities showed increasing rates of oxacillin-resistant *CoNS* (60.4%), piperacillin-tazobactam-resistant Gram-negative bacteria (26.5%), resistance of Gram-positive bacteria to vancomycin (<1%) and Gram-negative microorganisms to meropenem (1.25%).

Furthermore, Ceftriaxone was a predominant antibiotic utilized as monotherapy (23.4%); whereas Ceftriaxone plus metronidazole was a common combination of antibiotics (42.4%) followed by Ceftriaxone plus Gentamicin (23.7%) of patients. Ciprofloxacin (33.8%), Cloxacillin (16.2%), and Amoxicillin/Clavulanate (12.2%) were common PO antibiotics utilized. However, a study done in South Africa [5], the results of treatment show that the combinations of meropenem plus vancomycin and piperacillin-tazobactam plus amikacin plus vancomycin were used. In addition, oral combinations were co-amoxiclav plus ciprofloxacin (61%) and co- trimoxazole plus ciprofloxacin (61%). The reason for variation could be due to differences in isolated etiology and MSKIs type (only osteomyelitis was included in study of SA).

In the present study, (46.9%) of participants had treatment failure. The result agrees with a prior studies conducted in France 39% [22], India 37.5% (50), Latvia 47% [24], literature review on PJI in USA up to 50% [25] and South Africa 48% [26]. But, this finding was higher than previous studies done in India 23.3% [17], Switzerland 25.9% [27], South Africa 13% [26] and Canada 24.8% [28] respectively. Meanwhile, other studies from Nigeria [29] and Sudan [30] were reported a higher frequency of treatment failure outcomes 55% and 56.9% respectively, also in Minnesota 60% [31]compared to the present findings.

The variance in treatment outcome might be due to the presence of drug-resistant strains and late presentations of the patient for seeking medical care were higher in the present study. Besides, inclusion of a wide range of MSKIs types (but a study conducted in Sudan included only diabetic foot osteomyelitis and PJI in USA), prior antibiotic exposure before admission and the use of ceftriaxone in the majority of patients could also contribute to treatment failure in our study. The other possible explanation might be due to differences in the given treatment protocol from those studies and comorbidities (In the study of Nigeria, most of the patients had sickle cell anemia).

The identified predictor of treatment outcomes in this study are: being male, having microbiological growth, ceftriaxone resistance, presence of comorbidities, complications, antibiotics switching from IV to PO and surgical interventions.

Male patients were over twice more likely to develop treatment failures as compared to female patients. Our finding is consistent with a study conducted in Switzerland [27] and a systematic review and meta-analysis study done in the USA [32]. They state male patients were more likely to experience treatment failure than females. But another report from Switzerland revealed that being female is associated with poor clinical outcomes [33]. This could be due to patient characteristics (comorbidities, age, and occupations) or might be due to the majority of participants in most studies are male and difference in age of the studied population (in the study of Switzerland, participants were children). In fact, trauma is also a risk factor for MSKIs in the majority of male patients in the current study; as a result, they stay in the hospital for prolonged periods and are more prone to develop in hospital complications.

Having microbial growth on the culture result was also an independent predictor of treatment failure; patients with microbial growth had a threefold higher risk of treatment failure than patients without microbial growth. The current finding was similar to the report of the studies conducted in England (38), India [27], Portugal [33], and France [22], which found that patients who had microbial growth had a high risk of treatment failures as compared to those who had no growth. However, it was inconsistent with the study conducted at Pennsylvania (patients who had no growth had a high risk of treatment failure as compared to those patients who had microbial growth) [34].

These discrepancies could be the association of treatment failure with microbial growth is infections caused by more virulent organisms, which are more difficult to treat than those have been caused by less virulent organisms. While a possible explanation for culture-negative treatment failure might be premature antimicrobial therapy before the culture test was obtained, variance in the infecting organism and differences in MSKIs types (only PJI) is included in the study of Pennsylvania.

Similarly, ceftriaxone resistance was the other determinant of treatment failure. Patients who were resistant to ceftriaxone are at more than four times at risk of treatment failure than patients who were sensitive to ceftriaxone therapy. This was coherent with a study conducted in Ethiopia [35]. However, this finding was inconsistent with a study conducted in the USA [36]; the report states infections treated with ceftriaxone have a better-recovered outcome than vancomycin- treated infections. This inconsistency could be due to ceftriaxone sensitivity to most isolated etiologies in the study of USA and epidemiological antibiogram variation. While in our study, ceftriaxone was resistant to most of the identified etiologies. Also, differences in MSKIs type (only osteomyelitis is included in study of USA) might be a reason for the inconsistency.

Additionally, comorbidity was found to be an independent predictor of treatment failure in patients diagnosed with MSKIs. Patients with co-morbidity were about two times more likely to develop treatment failure than those without comorbid medical conditions. The current finding was supported by studies done in France [37], India [17], Spain [38], Switzerland [32], and India [39]. But, an opposing finding was reported from France [40], which states that no single comorbid condition was significantly associated with treatment failure outcomes. The discrepancy might be due to a difference in comorbidity types in France; the listed comorbidities are: neurological and/or psychiatric diseases, alcohol abuse, and fever. In other comparator studies, diabetes mellitus was the most common.

In this study, IV antibiotics switching and surgical interventions were protective against treatment failures among patients treated for MSKIs. Patients whose IV antibiotics were switched to PO antibiotics had seventy percent more likely to have recovered outcomes as compared to patients whose their antibiotics were not switched. The result is comparable to a previous study reported from USA revealed that an excellent outcome was seen after IV therapies were switched to oral antibiotic therapy [41]. In line with this, previous literatures conducted in Switzerland [32], England [42], and France [43] showed the non-inferiority of early oral step-down compared with prolonged IV therapies. However, it is contrary to study done USA; the finding showed treatment failure in the oral antibiotic group participants [44].

The inconsistency could be due to differences in MSKIs type studied, lower rate usage of timely surgical intervention with antibiotics, and variation in presence of comorbidities (most of the patients had peripheral osteomyelitis) in the USA report. But, in our study, variety types of MSKIs were included. And also, for the majority of our patients, surgical intervention was given with antibiotics.

Surgical intervention was another protective measure against treatment failures in MSKIs patients. Patients who underwent surgical procedures as treatment of MSKIs were about ninety percent more likely to have recovered treatment outcomes than patients who didn’t undergo surgical interventions. This result was comparable with other studies done in Ethiopia [6], Spain [45], India [24] and Switzerland [32]. However, a previously conducted study in Switzerland [46]; partially agreed with the requirement for surgical intervention in the management of MSKIs due to differences in MSKIs type that need surgical intervention). On the other hand, a Study conducted in USA [47] revealed providing surgical procedure was associated with treatment failure. While other study which is conducted in China [48], there was no significant difference in recovery outcome between groups having surgical intervention and hadn’t.

The possible explanation for the discrepancy might be due to differences in study population (In study of USA pediatrics are the studied participants), MSKIs type (only septic arthritis was included in study of China), the presence of comorbidity, and the presence of complications that requires surgical intervention. The observed recovered outcome for the present study could be on admission; half of the participants were presented with abscess, for which incision and drainage of the abscess were done, as well as debridement of infected tissues, irrigation, and removal of infected orthopedic prostheses were done. Those surgical interventions had a role in rapidly decreasing the bacterial load and avoiding potential complications.

In current study, patients who had treatment failures were developed at least one complication of musculoskeletal infections during their hospital stay. Systemic infection (27.2%), Deep vein thrombosis (25%), bone abscess (19.6%), amputation (7.6%), bone necrosis (6.5%), Knee Stiffness (5.4%), joint dysfunction (4.3%), and ankle stiffness (4.3%) were the identified in hospital complications type among patients with MSKIs. This finding was parallel to the report of studies conducted in Ethiopia [6] and South Africa [26]. While this pattern of complication was different from the results of the study conducted in Portugal (61), Ecuador [36], Brazil [49], and Thailand [49], which show the identified complications were arthrofibrosis, sepsis, pulmonary embolism, death, myositis, subluxation, pathological fracture and liver injury.

The inconsistency might be due to differences in etiology (fungal infections are cause of MSKIs) in Thailand, longer follow-up duration, presence of a large number of drugs susceptible microorganisms, difference in MSKIs risk factors and anatomical site involvement in Portugal, also differences in management protocol used at different institutions may have a role for the variation seen. But in our study, most of the identified etiology was resistant to the commonly used antibiotics in the setting, as well as due to short-term follow-up duration, long-term complications (i.e., Death) were not reported. Moreover, in low-income countries like Ethiopia, lack of equipment and materials necessary to maintain strict aseptic conditions and poor hygiene of patients could be a reason for high number of systemic infections.

The mean ± SD length of hospital stay for the present study was 38.74±13.17 days. This result was comparable to the findings of a study conducted in Tunisia that showed hospital stays of

38.4 days [50]. But, it was higher than the hospital stays in the UK (30), France [51], China [52], Kazakhstan [53] and India [54], which were 13, 21, 8.3, 17.5, and 26.9 days, respectively. The reason for longer hospital stays might be due to the presence of microorganisms that are associated with treatment failure. In addition, for most of our study participants, the cause of their MSKIs was trauma, especially bullet injury and road traffic accidents, since it may take them a longer period to heal. Moreover, 46.9% of enrolled patients develop in-hospital complications, and the majority of patients underwent surgical intervention, which may be a reason for prolonged hospitalization.

## Conclusion

This study highlights the significant burden of musculoskeletal infections (MSKIs) among adult patients at Jimma Medical Center, Ethiopia, with nearly half of the patients experiencing treatment failure. *Staphylococcus aureus* emerged as the most prevalent bacterial pathogen, demonstrating high resistance to commonly used antibiotics such as ceftriaxone. The findings underscore the critical importance of timely and appropriate management strategies, including the early transition from parenteral to oral antibiotics and prompt surgical interventions, which were shown to significantly improve treatment outcomes. Factors such as male sex, microbial growth, ceftriaxone resistance, comorbidities, and complications were identified as key predictors of treatment failure, while surgical intervention and antibiotic switching were protective against poor outcomes. These insights emphasize the need for tailored treatment protocols, enhanced antimicrobial stewardship, and improved infection control measures to address the growing challenge of antimicrobial resistance and optimize patient care in resource-limited settings. Future research should focus on longitudinal studies to further explore the long-term outcomes and cost-effectiveness of these interventions in similar contexts.

## Abbreviations

AST: Antimicrobial Susceptibility Tests
BJI: Bone and Joint Infection
BMI: Body mass Index
CBC: Complete blood count
CoNS: Coagulase-Negative Staphylococci
CRP: C-reactive protein
DVT: Deep vein thrombosis
ESR: Erythrocyte sedimentation rate
GNB: Gram-Negative Bacteria
IV: Intravenously
JMC: Jimma Medical Center
MRSA: Methicillin-resistant Staphylococcus aureus
MSKI: Musculoskeletal Infection
MSSA: Methicillin - Sensitive Staphylococcus aureus
NIRI: Non-Implant Related Infections
OAI: Osteoarticular Infection
OM: Osteomyelitis
OIRIs: Orthopedic implant-related infections
PJI: Periprosthetic joint infection
SA: Septic arthritis
SSIs: Surgical Site Infections
SSTIs: Skin and Soft-tissue Infections
